# A Prognostic Survival Model Incorporating Patient-Reported Outcomes for Transplant-Ineligible Patients With Multiple Myeloma

**DOI:** 10.1093/oncolo/oyae041

**Published:** 2024-04-18

**Authors:** Hira Mian, Hsien Seow, Amaris K Balitsky, Matthew C Cheung, Anastasia Gayowsky, Jason Tay, Tanya M Wildes, Arleigh McCurdy, Alissa Visram, Irwindeep Sandhu, Rinku Sutradhar

**Affiliations:** Department of Oncology, McMaster University, Hamilton, ON, Canada; Department of Oncology, McMaster University, Hamilton, ON, Canada; Department of Oncology, McMaster University, Hamilton, ON, Canada; Department of Medicine, Sunnybrook Health Sciences Centre, University of Toronto, Toronto, ON, Canada; ICES, McMaster University, Hamilton, ON, Canada; Department of Medicine, University of Calgary, Calgary, AB, Canada; Department of Medicine, University of Nebraska Medical Center/Nebraska Medicine, Omaha, NE, USA; Department of Medicine, The Ottawa Hospital Research Institute, University of Ottawa, Ottawa, ON, Canada; Department of Medicine, The Ottawa Hospital Research Institute, University of Ottawa, Ottawa, ON, Canada; Department of Medicine, University of Edmonton, Edmonton, AB, Canada; Institute for Health Policy, Management and Evaluation, University of Toronto, Toronto, ON, Canada; Division of Biostatistics, Dalla Lana School of Public Health, University of Toronto, Toronto, ON, Canada

**Keywords:** multiple myeloma, prognostic tool, patient-reported outcomes, survival

## Abstract

Developing prognostic tools specifically for patients themselves represents an important step in empowering patients to engage in shared decision-making. Incorporating patient-reported outcomes may improve the accuracy of these prognostic tools. We conducted a retrospective population-based study of transplant-ineligible (TIE) patients with multiple myeloma (MM) diagnosed between January 2007 and December 2018. A multivariable Cox regression model was developed to predict the risk of death within 1-year period from the index date. We identified 2356 patients with TIE MM. The following factors were associated with an increased risk of death within 1 year: age > 80 (HR 1.11), history of heart failure (HR 1.52), “CRAB” at diagnosis (HR 1.61), distance to cancer center (HR 1.25), prior radiation (HR 1.48), no proteosome inhibitor/immunomodulatory therapy usage (HR 1.36), recent emergency department (HR 1.55) or hospitalization (HR 2.13), poor performance status (ECOG 3-4 HR 1.76), and increasing number of severe symptoms (HR 1.56). Model discrimination was high with C-statistic of 0.74, and calibration was very good. To our knowledge, this represents one of the first prognostic models developed in MM incorporating patient-reported outcomes. This survival prognostic tool may improve communication regarding prognosis and shared decision-making among older adults with MM and their health care providers.

## Teaser

This article reports a prognostic model that was developed to predict survival in older patients with transplant-ineligible multiple myeloma and may improve communication regarding prognosis and shared decision-making.

**Implications for Practice**

Results of this study represent one of the first prognostic models incorporating patient-reported outcomes developed for multiple myeloma. This survival prognostic tool may improve communication regarding prognosis and shared decision-making among older adults with multiple myeloma and their health care providers.

## Introduction

Multiple myeloma (MM), a neoplasm characterized by the clonal proliferation of malignant plasma cells, is associated with major morbidity and mortality. The median age at diagnosis is 70 years, making MM a disease of older transplant-ineligible (TIE) adults.^1^ Although the outcomes for this cohort have improved over time, they still lag behind those in younger, transplant-eligible patients, with high rates of early mortality and symptom burden reported in this patient population.^2,3^

A number of prognostic tools have been developed in MM, including international myeloma staging system (ISS), the revised international myeloma staging system (R-ISS), and additional stratification tools that incorporate more complex genetic and molecular markers.^4-7^ While these tools are helpful in providing prognostic information, they are limited by the following: (1) these tools are mainly developed to be used by oncologists and health care providers, (2) these tools consist of specialized blood tests and/or cytogenetics test results which may not be readily available or interpretable to patients, (3) they are often used only at the time of diagnosis, and (4) do not take into account how variables including disease characteristics, treatments received, or comorbidities may impact prognosis.

Patients have a strong desire to understand their prognosis yet up to 60%-80% of patients have a discordant understanding of their prognosis compared to their oncologists.^8,9^ Lack of prognostic information or discordant prognostic information is associated with multiple negative outcomes, including receiving possibly futile treatments, delayed and lower utilization of palliative care, and reduced quality of life.^10,11^ Understanding this prognostic information is important for older adults with MM in making personalized decisions regarding planning for future personal, and health care needs. Therefore, there is also a need to develop patient-friendly prognostic tools that allow patients to access this information more readily and engage in shared decision-making with their oncologists.

In this study, we aimed to develop and validate a prognostic model to predict survival in patients with TIE MM. To ensure patients would be able to access this tool, we used clinical information and patient-reported outcomes (ie, self-reported symptom burden and functional status) that could be easily obtained or completed by patients. The objective of this prognostic model is to provide patients with TIE MM prognostic information which may allow them to engage in further conversation regarding their future needs and goals with their health care team.

## Methods

### Study Design and Population

Multiple administrative health care databases in the universal, single-payer, publicly funded system in Ontario, Canada were linked using a unique encrypted patient identifier and analyzed at ICES (formerly known as the Institute for Clinical Evaluative Sciences). The study was approved by the ethics committee of McMaster University and followed data confidentiality and privacy guidelines of ICES.

All adults (age ≥ 18) with a new diagnosis of MM (International Classification of Diseases for Oncology, 3rd Edition, histology code 9732) between January 2007 and December 2018 were identified. In the Canadian landscape, the decision to transplant is made at the time of diagnosis due to the subsequent different publicly funded treatment pathways for transplant eligible and TIE respectively. However, as there are no specific diagnostic codes that would allow us to distinguish between transplant-eligible and TIE patients, patients with TIE MM were defined as patients who did not receive a transplant in the 1 year following MM diagnosis. The treatment algorithm for the management of TIE MM is largely uniform in Canada due to the availability of publicly reimbursed therapies which consisted of either a proteosome inhibitor (PI) or immunomodulatory drug (IMID) consistent with funding algorithms.^12^ This includes a fixed-duration PI-based regimens like bortezomib-melphalan-dexamethasone for a total of 8-12 cycles which has been funded since 2009. Additionally, starting in 2017, lenalidomide/dexamethasone was available in first line for this patient population as well. While combination treatments such as lenalidomide/bortezomib/dexamethasone (RVd) or daratumumab/lenalidomide/dexamethasone have been funded since 2020 and 2022, respectively, those patients were not included in this study due to the time frame during which it was conducted. This 1-year mark following MM diagnosis was defined as the initial index date (year 1 model).

We subsequently used the Symptom Database to identify patients with TIE MM who had completed patient-reported symptoms (at least one symptom score completed in preceding 6 months). The Symptom Database started in 2007 when Cancer Care Ontario mandated the systematic screening of outpatients with cancers for symptoms using the Edmonton Symptom Assessment System (ESAS). ESAS is a validated and reliable patient-reported outcome tool that is used to assess 9 common cancer-associated symptoms, including pain.^13^ Patients report the severity of symptoms from 0 to 10 with severe symptoms defined as those scoring 7 or higher.

### Data Sources

We used a number of linked administrative databases (Supplementary Table S1): patient comorbidities were defined using previous health care encounters including hospital discharge summaries. Patient self-reported symptoms were captured using the Symptom Management Database as described above. Self-reported performance status of each oncology outpatient was recorded using the Palliative Performance Scale (PPS),^14^ a modified Karnofsky Performance Scale. The ECOG PS is comparable to and highly correlated with the clinician-reported PPS.^15,16^

### Outcomes

The primary outcome was 1-year all-cause mortality risk from the index date. All-cause mortality versus mortality specifically attributed from MM itself was picked as the outcomes as it was felt to be more relevant from a patient’s perspective.

The initial index date for the year 1 model was defined as 1 year following diagnosis (consistent with population inclusion criteria for the cohort, ie, no transplant within 1 year following diagnosis). Because information on patient-level characteristics and treatments changes over time, we also aimed to predict conditional survival probability among patients who were alive at the 2-year mark following diagnosis. This was done by moving the index date to the 2-year mark (year 2 model).

### Covariates

Each model included the following baseline covariates: age, sex, distance from cancer center (>50 km or more), MM-related factors (“CRAB” criteria^17^ at diagnosis, used only in the year 1 model), comorbidities (within 5 prior years), previous history of cancer in the last 15 years, low blood counts (hemoglobin < 100 g/L, platelets < 100 × 10^9^/L, and neutrophils < 1.0 × 10^9^/L), radiation (within 12 months prior), IMID/PI drug usage (within 12 months prior), hospitalization or emergency department visit (≥1 occurrence), functional status, and the number of symptom categories that were recorded as severe symptoms (ESAS score of ≥7). All of the above variables were felt to be easily accessible for patients or known by patients themselves. Self-reported performance status and symptoms burden were the 2 patient-reported outcomes included among the covariates. Unless otherwise indicated all covariates were measured within 6 months prior and up to 3 months post-index date.

### Statistical Analysis

Our primary outcome was 1-year mortality risk from the index date. Time-to-event models were used at each index date (1 and 2 years following diagnosis) for each new model.

#### Developing the Prediction Model

A total of 75% of the eligible patients were randomly selected for model derivation and 25% of the remaining patients were set aside for validation. The baseline characteristics between the derivation and the validation cohort were assessed to ensure there was random sampling.

First, using the derivation cohort only, a multivariable Cox proportional hazards regression model with baseline covariates was used to predict the risk of death at 1 year post-index date. A backward stepwise selection procedure with a liberal 2-sided *P*-value of <.10 was used to retain variables. Given the known impact of chronological age on survival, chronological age was included a priori in the model. Missing data from variables were handled by creating a missing data category for applicable variables.

#### Validating the Prediction Model

After the final regression model was created from the derivation cohort, the estimated 1-year probability of death (from the index date) was calculated for each patient in the validation cohort. This calculation was based on a combination of the patient’s-specific profile of covariate values, the model’s regression parameter estimates, and the baseline hazard function estimate. To assess model calibration (how close the model’s estimated risk is to the observed risk) in the validation cohort, the individual predicted 1-year mortality risks were ranked and then binned into deciles, after which the average predicted risk was plotted against the observed risk within each decile. Points closer to the 45° line indicate better calibration. We also measured the discrimination of the model (ability of the model to distinguish between a patient who had died from one who did not die) in the validation index using the concordance index (C-index). The primary outcome and the statistical plan were prespecified and done as per previous studies done by our group.^18^ All analyses were conducted using the statistical software R, version 2.15 and SAS version 9.

## Results

**Figure 1.**
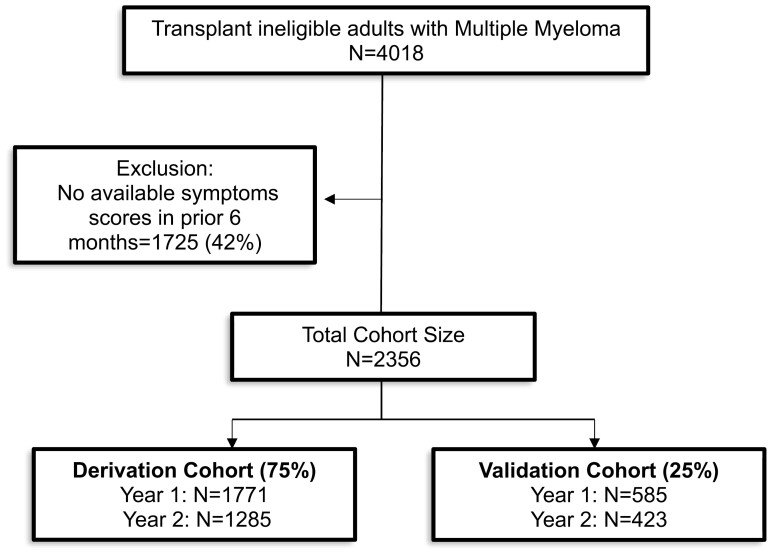
Cohort selection.

We identified a total of 2356 TIE adults with MM who had completed symptom assessment scores and therefore included in our cohort. Of note, only a small proportion of patients had a stem cell transplant 1 year following the index date (*n* = 100/2356, 4.2%) till the date of last follow-up or death consistent with our population being a TIE cohort. In the subsequent year (year 2), this number decreased to a total of 1708 patients (Fig. 1).

Baseline characteristics of patients with TIE NDMM in years 1 and 2 are outlined in Table 1. In the derivation cohort in year 1, the median age of the patients was 75 with over 25% of the patients of age 80 years or older. With regards to previous comorbidities, a history of congestive heart failure (15.2%) and hypertension (13.4%) were the 2 most commonly recorded comorbidities. A total of 18.1% of the patients had another cancer in the 15 years preceding. There were high rates of health care utilization with 33.1% and 51.7% of patients experiencing an inpatient hospitalization or emergency department visit. With regards to treatment, 89.8% of the patients had received a PI/IMID. With regards to functional status, the majority of patients (58.7%) had an ECOG PS of 0-1, with 17.2% ECOG 2 and 8.2% ECOG 3-4. With regards to symptom burden, out of a possible 9 categories of self-reported symptoms (ie, pain, depression, well-being, shortness of breath, anxiety, nausea, tiredness, drowsiness, and appetite with severe symptoms defined as a score of 7 or higher out of 10), 34.4% of the patients reported severe symptoms in 1-3 categories, 15.1% in 5-6 categories, and 6.3% reported in 7-9 categories.

**Table 1.** Baseline characteristics of the cohort for the years 1 and 2 model.

Variable	Value	Year 1 (*N* = 2356)		Year 2 (*N* = 1708)	
		Derivation	Validation	Derivation	Validation
		*N* = 1771	*N* = 585	*N* = 1285	*N* = 423
Age (years)	Median (IQR)	75 (70-80)	75 (70-80)	76 (71-80)	76 (71-81)
	*N* (%), <65	189 (10.7)	66 (11.3)	115 (9.0)	35 (8.3)
	*N* (%), 65-70	262 (14.8)	83 (14.2)	169 (13.2)	65 (15.4)
	*N* (%), 71-79	827 (46.7)	272 (46.5)	629 (49.0)	189 (44.7)
	*N* (%), ≥80	493 (27.8)	164 (28.0)	372 (29.0)	134 (21.7)
Sex	Female	745 (42.1)	250 (42.7)	542 (42.2)	203 (48.0)
	Male	1026 (57.9)	335 (57.3)	743 (57.8)	220 (52.1)
Distance ≥ 50 km to cancer center	No	1417 (80.0)	451 (77.1)	1002 (78.0)	346 (81.8)
	Yes	353 (19.9)	134 (22.9)	280 (21.8)	76 (18.0)
Comorbidities (5 years prior)	Asthma	48 (2.7)	9 (1.5)	29 (2.3)	11 (2.6)
	CHF	270 (15.2)	96 (16.4)	207 (16.1)	61 (14.4)
	COPD	108 (6.1)	35 (6.0)	83 (6.6)	21 (5.0)
	Hypertension	238 (13.4)	83 (14.2)	163 (12.7)	56 (13.2)
	Diabetes	119 (6.7)	42 (7.2)	84 (6.5)	24 (5.8)
	MI	131 (7.4)	57 (9.7)	112 (8.7)	30 (7.1)
Previous other cancer (15 years prior)		321 (18.1)	116 (19.8)	241 (18.6)	78 (18.4)
CRAB (within 6 months of diagnosis)		610 (34.4)	209 (35.7)	N/A	N/A
Hemoglobin < 100g/L (6 months prior)		663 (37.4)	212 (36.2)	448 (34.9)	134 (31.7)
Platelets < 100 × 10^3^/µL (6 months)		589 (33.3)	182 (31.1)	305 (23.7)	90 (21.3)
Neutrophil < 1.0 × 10^3^/µL (6 months)		225 (12.7)	72 (12.3)	158 (12.3)	53 (12.5)
Hospitalization (6 months prior)		587 (33.1)	172 (29.4)	347 (27.0)	109 (25.7)
ER visit (6 months prior)		916 (51.7)	324 (55.4)	576 (44.8)	184 (43.5)
Radiation (12 months prior)		558 (31.5)	177 (30.3)	133 (10.4)	46 (10.9)
IMID/PI drugs (12 months prior)		1590 (89.8)	538 (92.0)	611 (47.6)	207 (48.9)
Functional status (worse score in 6 months prior)	0/1	1040 (58.7)	345 (59.0)	821 (63.9)	280 (66.2)
	2	305 (17.2)	92 (15.7)	179 (13.9)	57 (13.5)
	3/4	145 (8.2)	57 (9.7)	91 (7.1)	28 (6.6)
	Missing	281 (15.9)	91 (15.6)	194 (15.1)	58 (13.7)
Number of symptom categories with severe symptoms	0	782 (44.2)	265 (45.3)	725 (56.4)	240 (56.7)
	1-3	609 (34.4)	195 (33.3)	359 (27.9)	113 (26.7)
	4-6	268 (15.1)	92 (15.7)	150 (11.7)	51 (12.1)
	7-9	112 (6.3)	33 (5.6)	51 (4.0)	19 (4.5)

Abbreviations: CHF, congestive heart failure; COPD, chronic obstructive pulmonary disease; CRAB, hypercalcemia renal failure, anemia, and bone disease; ECOG, Eastern Cooperative Oncology Group; ER, emergency department; IQR, interquartile range; IMID, immunomodulatory drug; MI, myocardial infarction; PI, proteosome inhibitor.

Data are presented as number (percentage) unless otherwise indicated.

If covariates values were missing, we also included any variables up to 3 months post-index date unless otherwise indicated.

In year 2 derivation cohort, similar trends were noted with the exception that there were fewer patients receiving radiation or IMID/PI drugs (common treatment regimens during the study time frame included fixed-duration PI-based regimens and therefore many patients were not on any treatment during year 2, as expected). There were also lower rates of health care utilization including hospitalization (27.0%) and emergency department visits (44.8%) in the preceding 6 months. With regards to functional status, there was a lower proportion of patients with ECOG 2 or ECOG 3-4 in year 2 as compared to the year 1 derivation cohort. Similarly, there was a smaller proportion of patients with >1 severe symptom as compared to year 1.

**Figure 2.**
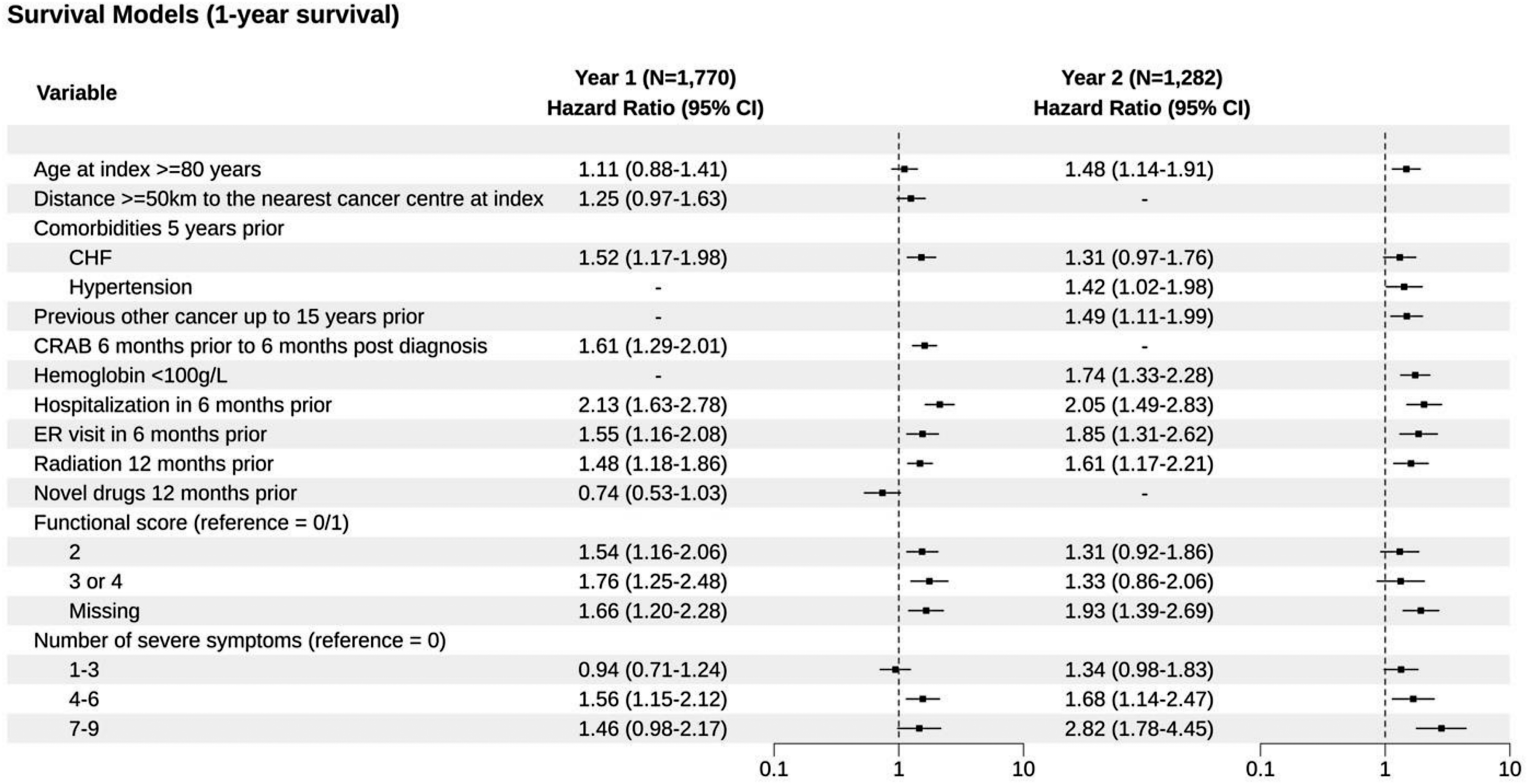
Final models for survival at 1 year for the derivation cohorts for the years 1 and 2 models. Abbreviations: CHF, congestive heart failure; CRAB, hypercalcemia renal failure, anemia, and bone disease; ECOG, Eastern Cooperative Oncology Group; ESAS, Edmonton Symptom Assessment Scale (included those with high ESAS ≥ 7 score corresponding with severe symptoms); ER, emergency department; IQR, interquartile range.

Two distinct models were created following stepwise backward selection for years 1 and 2 (Fig. 2). As each model was independently created, there was a slightly different set of variables that were included in the final model in years 1 and 2. In year 1, the following factors were associated with an increased risk of death: age > 80, distance > 50 km from the nearest cancer center, prior history of congestive heart failure, myeloma-defining CRAB symptoms present at diagnosis, hospitalization/ER visit in 6 months prior, receipt of radiation, poor functional status, and a high symptom burden as indicated by an increased number of severe symptoms. IMID/PI drug receipt was associated with a lower risk of death.

The year 2 model yielded similar variables and overall trends with a few exceptions. Distance to the nearest cancer center, CRAB symptoms, or receipt of IMID/PI drugs did not meet the statistical threshold to be included in the year 2 model. Additional variables that were included only in the year 2 model were prior history of hypertension, prior history of other cancers, and a hemoglobin level of <100 g/L.

**Figure 3.**
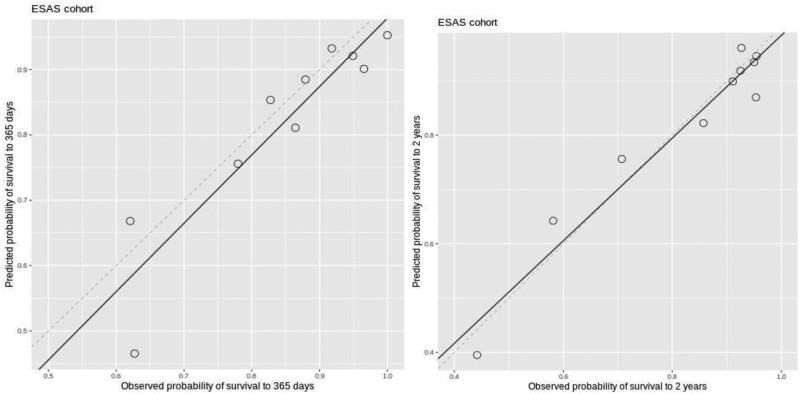
Calibration plot for years 1 and 2 survival model.

With regards to our model fit, calibration plots were created for the years 1 and 2 model (Fig. 3). Calibration for the year 1 model is good, as the points are near the 45° line; and calibration for the year 2 model is better as the fitted dotted line is closer to the 45° line. Overall, model discrimination was high with the c-index being 0.74 (year 1) and 0.73 (year 2 model).

To highlight how these models would work, a hypothetical scenario is presented. Mr. BT, a 75-year-old male with MM received treatment in the year prior with a PI drug regimen—RVd. He lives in the countryside with a nearest cancer center being >50 km away. This patient recalls presenting with a fracture at the time of diagnosis. He has no known comorbidities. He did not receive any radiation and did not have any emergency department or hospital admission this year. He is functionally active farming but does endorse feeling pain that can be severe at times at the site of his previous fracture. Using this model (year 1), the probability of surviving another 1 year would be 91.3%. By year 2, although he is still doing well, he experienced a recent hospital admission for pneumonia and finds that although he is able to take care of himself, he is unable to participate in the same farming activities. He now endorses at least 4 symptoms that can be quite severe at times including pain, lack of energy, poor overall well-being, and depressed on occasion. His bloodwork including his hemoglobin remains normal. Using this model at year 2, the probability of Mr. BT surviving another year from this landmark time would be 83.5%.

In contrast, if Mrs. GT is an 83-year-old female with MM who received treatment in the year prior with a PI drug regimen—RVd. She lives close to a hospital with her family. She currently sees a cardiologist for congestive heart failure and high blood pressure. She has a previous history of breast cancer approximately 10 years ago which was treated with chemotherapy and radiation. With regards to her MM, she recalls presenting with kidney damage and bone pain at the time of diagnosis. As she had significant pain in her right arm at the time of diagnosis, she recalls going for radiation treatments. She developed pneumonia a few months ago while she was on treatment and was treated in the emergency department. She also unfortunately had one 5-day hospital admission for her heart failure a month ago during which her medications were changed. Ever since her hospital admission, she has spent most of her time sitting on her couch. She has ongoing severe pain but also has shortness of breath and fatigue ever since her admission. She is very worried about the future and often finds herself unable to enjoy the things she used to do previously. Her family has also noticed a decrease in her appetite and overall well-being in the last few months. Using this model (year 1), the probability of Mrs. GT surviving another 1 year would be 19.3%. By year 2, things have continued to deteriorate. She continues to have additional admissions to the hospital and developed another pneumonia requiring treatment in the emergency department. She has also needed a blood transfusion now in the last 2 months. She continues to experience all of the same symptoms and has not noticed any improvement in them over the year. Using this model at year 2, the probability of Mrs. GT surviving another year from this landmark time would be 1.8%.

## Discussion

Using a population cohort, we have developed and validated a survival model specifically for patients with TIE MM incorporating patient-reported outcomes. This model has the potential to be completed by patients themselves as it incorporates variables that can either be accessed or self-reported by patients including symptom burden. Furthermore, this model accounts for changes in variables, including patient, disease, and treatment characteristics, at different time points during the MM disease trajectory.

There are a number of prognostic tools that exist in MM. Conventional prognostic tools include the ISS^6^ and more recently the R-ISS which incorporates cytogenetics.^19^ Additional tools for prognostication in MM also exist which are tailored more specifically to either the patient’s functional status such as the International Myeloma Working Group Frailty Score^20^ or more specifically focused on additional disease characteristics such as the SKY92^7^ which on gene expression profiles in MM. While all the above tools are important in providing valuable prognostic information, they are often limited to being used only by health care providers. Cytogenetic results, for example, although extremely valuable may not be easily available or interpretable by patients themselves limiting their use for patient’s self-understanding of their prognosis. Additionally, outcomes like overall survival are influenced by a combination of specific patient, disease, and treatment characteristics and can change over time. Existing prognostic tools exclusively using MM parameters may not capture additional important information about the patient (ie, previous and/or changing comorbidities). Additionally, because our model is recalculated in the subsequent year (year 2), it can account for changing patient characteristics, compared to other prognostic tools that are created and validated at one specific time point, most commonly at the time of diagnosis.

Our model is also unique in that it incorporates patient-reported outcomes of symptom burden. Patient-reported outcomes have been increasingly important in oncology and particularly in disease like MM which are associated with high symptom burden.^3,21,22^ Patient self-reported measures have the advantage of potentially more accurately reflecting the function of a patient as this measure is less subject to health care provider bias and interpretation.^23^ Previous studies have demonstrated that patient-reported outcomes are prognostic of important outcomes including patient satisfaction, health care utilization, treatment toxicity, and overall survival.^24-27^ The province of Ontario in Canada instituted a province-wide patient self-reported assessment measure whereby patients complete the simple tool, ESAS, every month in outpatient clinics. Previous work by our group has shown the high symptom burden of patients with MM, which is associated with increased health care utilization.^26^ This current prognostic model builds upon our previous work and incorporates these self-reported patient outcomes in predicting overall survival.

To understand how our model may be used, we have presented a hypothetical scenario of how the model may be used by patients themselves by inputting variables that would be easily obtainable to be able to be self-completed by patients. Understanding the predicted 1-year survival risk may be useful for patient and health care team to initiate discussions around prognosis, goals of care, and any future planning with regards to involving supportive care and/or palliative care. Our model also demonstrates how risk may change over time. For example, in the year 2 model, the patient was hospitalized the year prior and was also experiencing additional symptoms. In this case, the subsequent 1-year survival decreased based upon the changing variables and may lead to further discussion among the patient, caregivers, and health care team regarding the overall outlook of their health trajectory. It is important to note that this tool should be used in discussion with health care teams as any prognostic information should be available to the patient with adequate supports in place to ensure patients are able to both understand the prognostic information and have the subsequent resources for any follow-up steps required. Therefore, the purpose of this tool is to incorporate changes in patient-specific characteristics including symptom burden, performance status along the MM disease trajectory to help initiate discussions and guide shared decision-making among patients, caregivers, and health care providers.

Our study has certain limitations. Our prognostic model has been created and validated in an Ontario database and therefore the results may not be generalizable to all settings including other jurisdictions with potential different access to therapeutics. Combination therapies with PI and IMID (ie, RVd) or anti-CD38-based therapies were not captured in this cohort as they were only recently approved in Ontario in this cohort and therefore we cannot elucidate the impact of these therapies on our model. Additionally, as this model relies on administrative databases, there are inherent limitations such as our inability to access MM disease stage, response to therapy or treatment toxicity which can impact prognosis. This also leads to a major limitation in our cohort as we are unable to evaluate this model compared to other prognostic scores including ISS, R-ISS, or more recently models incorporating gene expression profiling. We also chose to create this prognostic score specifically among patients with known symptom burden information; however, there are patients who chose not to fill out symptom burden assessment and therefore the prognostic value of this score among those patients is unknown. Lastly, although this prognostic model was validated, we have not conducted any feasibility testing of this model with patients or formatted this model into an easily accessible tool for patients, therefore, we have not yet incorporated patient feedback into the delivery of this information. The next step will be to work with user information specialists to potentially present this information in an easily available format (ie, online web-based version) and to continually update this model as new treatment modalities are introduced. Further qualitative studies will be needed on how to further improve this model using patient experience and input once this tool is used.

## Conclusion

We present the first-ever prognostic survival model for TIE patients with MM that specifically incorporates patient-reported outcomes. This tool has the potential to be used at different time points during the disease trajectory, accounting for longitudinal changes in patient, disease, and treatment characteristics. Overall, this tool has the potential to help facilitate discussions between patients, caregiver, and health care providers regarding prognosis including changes in it over time, ultimately allowing for improved shared decision-making during the MM disease trajectory.

## Supplementary Material

oyae041_suppl_Supplementary_Tables_1

## Data Availability

The data underlying this article are available in ICES Ontario Databases.

## Previous Presentation

This study was presented as an oral abstract at the 20th annual IMS meeting in Athens 2023.
