# Supplementary material for: A Prognostic Survival Model Incorporating Patient-Reported Outcomes for Transplant-Ineligible Patients With Multiple Myeloma
Source: Oncologist. 2024 Apr 18;29(6):519–26. doi: 10.1093/oncolo/oyae041 (PMC11145018; doi:10.1093/oncolo/oyae041)
Supplement: oyae041_suppl_Supplementary_Tables_1 [file oyae041_suppl_supplementary_tables_1.docx]

| Database |
| --- |
| Ontario Cancer Registry,  Activity Level Reporting  New Drug Funding Database (contains chemotherapy and radiation details), Discharge Abstract Database and National Acute Care Registry System (emergency department and hospital visit details)  Home Care Database  Registered Persons Database (RPDB)  Ontario Health Insurance Plan Claims Database (OHIP)  Ontario Drug Benefit Claims (ODB)  Postal Code Conversion File (PCCF)  Information about Ontario health care institutions funded by the Ministry of Health and Long-Term Care (INST)  Continuing Care Reporting System (CCRS)  Ontario Laboratories Information System (OLIS)  Ontario Asthma dataset (ASTHMA)   Congestive Heart Failure (CHF)  Ontario Hypertension dataset (HYPER)  Ontario Diabetes Dataset (ODD)  Symptom Management Database |

**Supplementary Table 1:** List of linked administrative databases in Ontario, Canada utilized in this project
